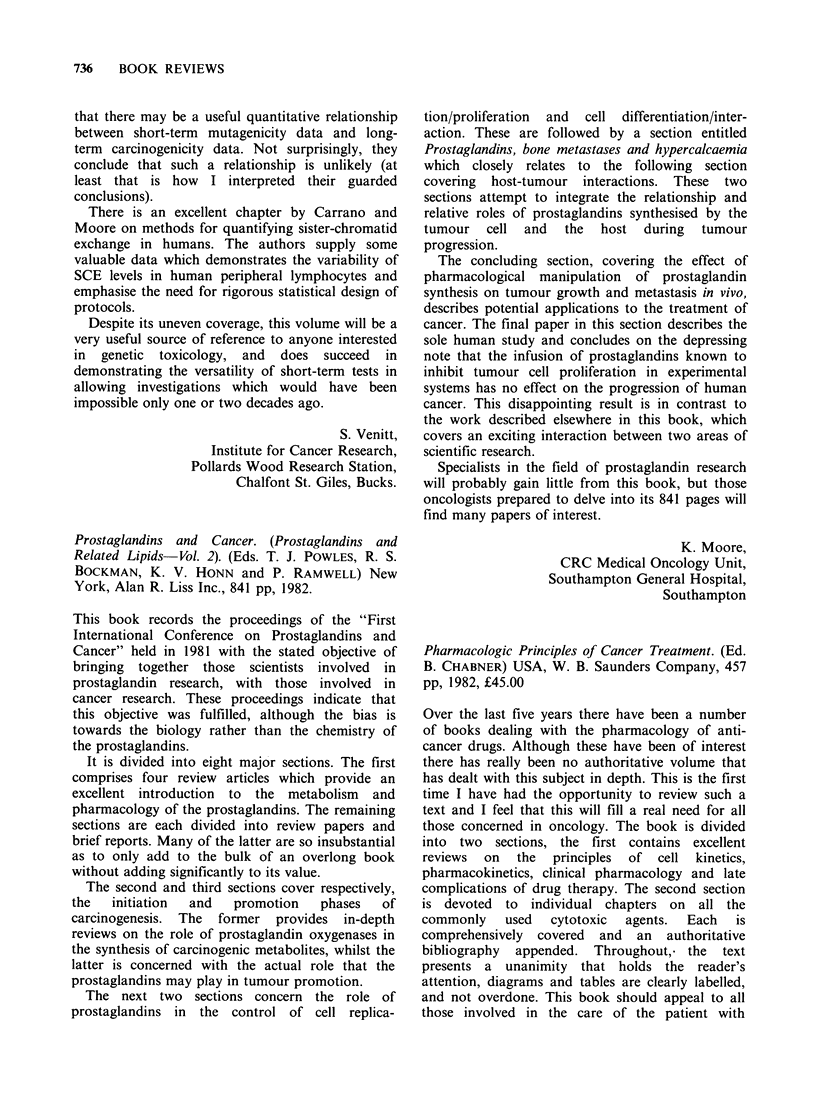# Prostaglandins and Cancer

**Published:** 1983-05

**Authors:** K. Moore


*Prostaglandins and Cancer.* (*Prostaglandins and Related Lipids—Vol. 2*). (Eds. T. J. POWLES, R. S. BOCKMAN, K. V. HONN and P. RAMWELL) New York, Alan R. Liss Inc., 841 pp, 1982.

This book records the proceedings of the "First International Conference on Prostaglandins and Cancer" held in 1981 with the stated objective of bringing together those scientists involved in prostaglandin research, with those involved in cancer research. These proceedings indicate that this objective was fulfilled, although the bias is towards the biology rather than the chemistry of the prostaglandins.

It is divided into eight major sections. The first comprises four review articles which provide an excellent introduction to the metabolism and pharmacology of the prostaglandins. The remaining sections are each divided into review papers and brief reports. Many of the latter are so insubstantial as to only add to the bulk of an overlong book without adding significantly to its value.

The second and third sections cover respectively, the initiation and promotion phases of carcinogenesis. The former provides in-depth reviews on the role of prostaglandin oxygenases in the synthesis of carcinogenic metabolites, whilst the latter is concerned with the actual role that the prostaglandins may play in tumour promotion.

The next two sections concern the role of prostaglandins in the control of cell replication/proliferation and cell differentiation/interaction. These are followed by a section entitled *Prostaglandins, bone metastases and hypercalcaemia* which closely relates to the following section covering host-tumour interactions. These two sections attempt to integrate the relationship and relative roles of prostaglandins synthesised by the tumour cell and the host during tumour progression.

The concluding section, covering the effect of pharmacological manipulation of prostaglandin synthesis on tumour growth and metastasis *in vivo,* describes potential applications to the treatment of cancer. The final paper in this section describes the sole human study and concludes on the depressing note that the infusion of prostaglandins known to inhibit tumour cell proliferation in experimental systems has no effect on the progression of human cancer. This disappointing result is in contrast to the work described elsewhere in this book, which covers an exciting interaction between two areas of scientific research.

Specialists in the field of prostaglandin research will probably gain little from this book, but those oncologists prepared to delve into its 841 pages will find many papers of interest.

K. Moore,
CRC Medical Oncology Unit,
Southampton General Hospital,
Southampton